# Evolution of CEACAM1 N Domain Biologically Active Sites in Primates

**DOI:** 10.3390/biology14121744

**Published:** 2025-12-05

**Authors:** Keith M. Skubitz, Wolfgang Zimmermann

**Affiliations:** 1 Department of Medicine, Division of Hematology, Oncology and Transplantation, University of Minnesota, Minneapolis, MN 55455, USA; 2 Masonic Cancer Center, University of Minnesota, Minneapolis, MN 55455, USA; 3 Immunology Laboratory, LIFE Center, Department of Urology, University Hospital, Ludwig-Maximilians University, D-80802 Munich, Germany; wolfgang.zimmermann@med.uni-muenchen.de

**Keywords:** neutrophil, CEACAM1, CEACAM3, CEACAM6, CEACAM8, CD66a, CD66b, CD66c, CD66d, CEA, peptide, evolution

## Abstract

CEACAMs are involved in a variety of physiological processes including cell adhesion, regulating the immune system, serving as entry receptors for a variety of pathogens, and regulating insulin receptor levels. Earlier studies identified five peptides from the N domain of human CEACAM1 that have stimulatory activity on human neutrophils. We compared the CEACAM N domain sequences, and also the amino acid sequences of the five CEACAM1 N domain peptides with biological activity in human neutrophils, among selected primates. Close similarity of the N domains was observed in the primates examined. The CEACAM1 N domains were more similar within great apes, Old World monkeys, New World monkeys, and lemurs/tarsiers than between groups. Differences in the amino acid sequences of some active peptides were observed among species; some differences are predicted to result in a loss of activity in the human neutrophil system. One amino acid change in the CD66a-1 peptide region that results in the loss of neutrophil activating activity in humans was observed in bonobos but not in the closely related chimpanzee which inhabits the opposite side of the Congo river. The same amino acid change was found to be a very rare event in humans. Changes in the CD66a-2 and CD66a-3 peptide regions were also observed in select human populations, some of which were differentially present in the chimpanzee and bonobo, as well as others that were not found in the other primates studied. In addition, a haplotype involving SNPs resulting in amino acid changes immediately adjacent to peptides CD66a-1, 3, and 7 were found in select human populations. Since CEACAM1 serves as a receptor for multiple infectious agents, selective pressure of an unidentified pathogen could be responsible for these differences. Given the diverse activities of CEACAM1 in humans, variant alleles in these domains might also have diverse effects in different populations.

## Simple Summary

Carcinoembryonic antigen-related cell adhesion molecules (CEACAMs) are involved in various processes, including cell adhesion, immune function, acting as pathogen receptors, and regulating insulin receptors. CEACAMs are recognized by a cluster of differentiation 66 (CD66) antibodies. Earlier studies identified five small regions from human CEACAM1 that activate neutrophils. If these regions are uniquely important, they may have evolved divergently in different species. We compared these small regions in primates. While overall the outer CEACAM1 region was very similar, differences in some active regions predicted to lack biological activity were observed among species. One amino acid change in one region that results in a loss of activity in humans was observed in bonobos but not in the closely related chimpanzees. The same change was found to be a rare event in humans. Changes in other regions were also observed in select human populations, some of which were differentially present among primates. In addition, changes involving amino acids immediately adjacent to some active regions were found in select human populations. We conclude that since CEACAM1 is utilized by a variety of pathogens as an entry receptor, selective pressure of an unidentified pathogen could be responsible for these evolutionary differences. In addition, given the diverse biological activities of CEACAM1 in humans, variant alleles in these domains might also have diverse effects in different populations that may modulate several disease processes.

## 1. Introduction

Carcinoembryonic antigen-related cell adhesion molecules (CEACAMs) belong to the immunoglobulin superfamily [1,2]. The human CEACAM gene family consists of 24 expressible members encoding transmembrane-bound members (CEACAM1, 3, 4, 18, 19, 20, 21) and glycosylphosphatidylinositol (GPI)-anchored proteins (CEACAM5, 6, 7, 8) as well as secreted members including the trophoblast-expressed pregnancy-specific glycoproteins (PSG1-12) and the inner ear-specific CEACAM16. CEACAMs are recognized by a cluster of differentiation 66 (CD66) antibodies. They are involved in a variety of physiological processes including cell adhesion, regulating the immune system, serving as receptors for a variety of pathogens, and regulating insulin receptor levels [3,4,5,6,7,8,9,10,11,12,13,14,15,16]. CEACAM1 is important for the regulation of CD4 T cell activation during inflammation probably via its immunoreceptor tyrosine-based inhibition motif (ITIM) [17]. This inhibitory property and its expression on epithelial cells have aided the independent appropriation of CEACAM1 as an entry receptor by a large number of mostly bacterial but also fungal pathogen species in humans (and probably other species) to gain access to the host and to concomitantly disable immune responses. CEACAM decoy receptors (CEACAM3, CEACAM5, CEACAM6) evolved in hosts as countermeasures, with pathogen-binding domains within the NH_2_-terminal immunoglobulin variable (IgV)-like or N domains that closely resemble those of CEACAM1 [16]. The neutrophil-specific human CEACAM3 decoy receptor diverts CEACAM1-binding pathogens to granulocytes and initiates the internalization and destruction of bacteria via its endocytic motif. CEACAM1 appears first in fish [18] and is found in all vertebrates including amphibians [19], reptiles, birds [Zimmermann and Kammerer, (submitted to BMC Genomics)], and mammals [20]. The role of CEACAM1 on neutrophils, where it is co-expressed with CEACAM3, 6 and 8, although in different compartments, is less clear [13]. In mice, CEACAM1 regulates granulopoiesis by inhibition of granulocyte colony-stimulating factor receptor [21].

Earlier studies have identified five peptides from the N domain of CEACAM1 that have stimulatory activity on human neutrophils including up-regulating or down-regulating cell surface receptors and increasing adhesion to endothelial cells [22,23,24]. Since the biological activity of CEACAMs likely evolved due to beneficial functional effects, and owing partly to selective pressure from pathogens, we examined whether these peptide regions might be conserved among selected primates.

We compared the CEACAM N domain sequences, and also the amino acid sequences of the five CEACAM1 N domain peptides with biological activity in human neutrophils, among selected primates. We observed close similarity of the N domains in the primates examined. Differences in the amino acid sequences of some active peptides were observed among species; some differences were predicted to result in loss of activity in the human neutrophil system. Some of the same amino acid changes were also found to be a very rare event in humans, primarily in populations from Africa and Asia. One set of three SNPs adjacent to active peptides were co-inherited as a haplotype in ~40–50% of some African tribes; each of these was found in chimpanzees and in bonobos. Since CEACAM1 serves as a receptor for multiple infectious agents, selective pressure of an unidentified pathogen could be responsible for these differences. Given the diverse activities of CEACAM1 in humans, variant alleles in these domains might also have diverse effects in different populations.

## 2. Materials and Methods

### 2.1. N Domain Amino Acid Sequences

N domain exon nucleotide sequences were retrieved using the NCBI BLAST/BLAT tools (http://www.ncbi.nlm.nih.gov/BLAST) and the Ensembl database (GRCh38.p14) and WGS contig databases using default parameters. Primate CEACAM1 sequences were accessed 17 April 2025. For identification of primate *CEACAM* and *PSG* genes, human N exon nucleotide sequences were used for the search. Orthologous *CEACAM* genes were identified by synteny (same order of CEACAM genes and presence of conserved neighboring genes as in the human CEACAM gene locus) and/or the physical linkage and expected distance of N exons and unique exons (e.g., exons encoding ITIMs or the CEACAM1-specific CEACAM1A2 domain) using sufficiently large contigs. The N domain encoding exons were translated using the Expasy Translate tool (https://web.expasy.org/translate/, accessed 17 April 2025).

### 2.2. Phylogenetic Analyses

Phylogenetic analyses based on mature N domain (with leader sequences removed), active peptide, and critical subregion amino acid sequences were performed and visualized with ClustalW (http://www.genome.jp/tools/clustalw, accessed 17 April 2025). Amino acid sequence alignments were performed using the KALIGN multiple protein sequence alignment tool (https://npsa.lyon.inserm.fr/cgi-bin/npsa_automat.pl?page=/NPSA/npsa_kalign.html, accessed 17 April 2025).

### 2.3. Identification of Single Nucleotide Polymorphisms (SNPs) in Human Populations

The position of missense CEACAM1 SNPs in N exons and their frequencies identified within the 1000 Genomes phase 3 project were retrieved using the Ensembl (https://www.ensembl.org/Homo_sapiens/Info/Index, accessed 17 April 2025) and the GnomADv4.1 (https://gnomad.broadinstitute.org/news/2024-04-gnomad-v4-1/, accessed 17 April 2025) databases. In Ensembl, these missense SNPs can be identified in the exon sequence displayed in the exon visualization mode as yellow highlighted nucleotide variants. Variants can be further explored by activating the “explore this variant” button in the associated dropdown menu. Information on SNP frequencies in various human populations can be obtained by activating the “Population Allele Frequencies” button in the dropdown menu. We included African (AFR), American (AMR), East Asian (EAS), European (EUR), and South Asian (SAS) populations in our analyses.

## 3. Results

### 3.1. N Domain Exon Nucleotide Sequences from Pathogen CEACAM Receptors Exhibit a Closer Relationship Within than Between Species in Humans and Other Primates

**Figure 1:**
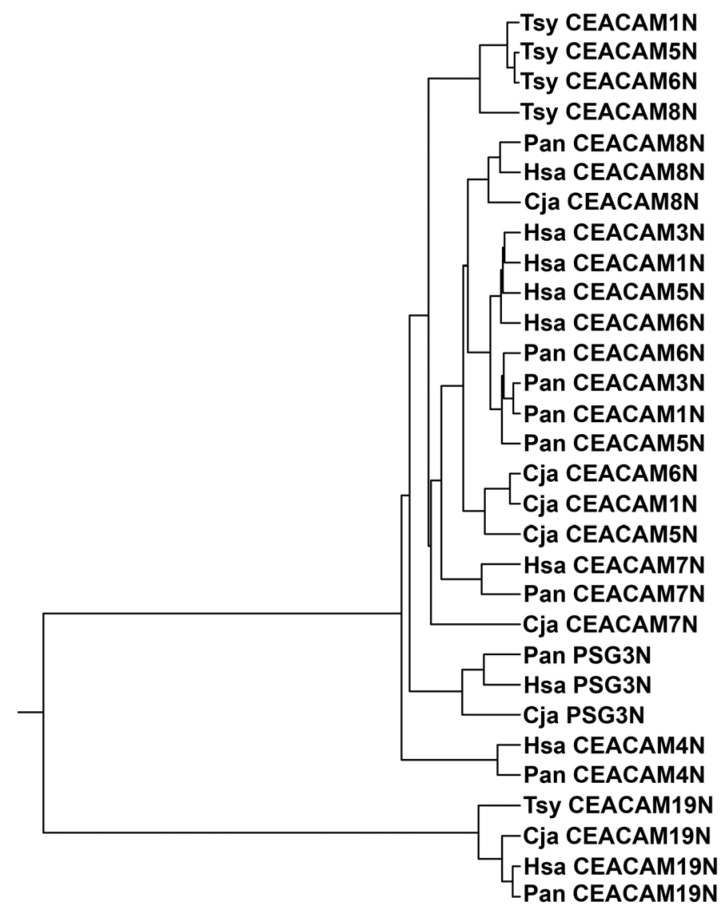
Phylogenetic tree of N exon nucleotide sequences of *CEACAM1*, *3*, *4*, *5*, *6*, *7*, *8*, and *19* and *PSG3*, one representative of the PSG subgroup, of selected primates. The phylogenetic relationship of the nucleotide sequences of the N domain exons of *CEACAM1* in various primate species is shown, where the length of the line reflects the similarity of sequences using the ClustalW technique, with shorter length representing higher similarity. Cja (*Callithrix jacchus*; marmoset, a New World monkey), Hsa (*Homo sapiens*; man, a great ape), Pan (*Papio anubis*; olive baboon, an Old World monkey), and the more distantly related Tsy (*Tarsius syrichta*; tarsier). PSG, pregnancy-specific glycoprotein.

Comparison of mature N domain amino acid sequences of CEACAM1, 3, 4, 5, 6, 7, 8, and 19, and pregnancy-specific glycoprotein (PSG) 3 (one representative of the PSG subgroup with highly similar members) of human and selected primates representing great apes [(human, Hsa), Old World monkeys (olive baboon, Pan) and New World monkeys (marmoset, Cja), as well as the more distantly related tarsius (Tsy)] (Figure 1 and Supplementary Figure S1) revealed that the N domains of human CEACAMs 1, 3, 5, and 6 are more closely related to each other in humans than to homologous domains in other species. In contrast, human CEACAM4, CEACAM8, and CEACAM19 N domains, which do not bind known pathogens [12,14], show more similarity with CEACAM4, CEACAM8, CEACAM19, and PSG3 N domains, respectively, in the other primates (Figure 1), thus exhibiting an orthologous relationship. A similar finding was observed with the baboon (Pan), an Old World monkey, and the marmoset (Cja), a New World monkey (Figure 1), in that the N domains of baboon CEACAMs 1, 3, 5, and 6 are more closely related to the homologous CEACAMs in the baboon than to the marmoset Cja (Figure 1) or the tarsier (Tsy). CEACAM3 and CEACAM4 do not exist in New World monkeys [4,15].

### 3.2. Phylogeny of Primate Mature CEACAM1 N Amino Acid Sequences Reflect Primate Relationships

**Figure 2:**
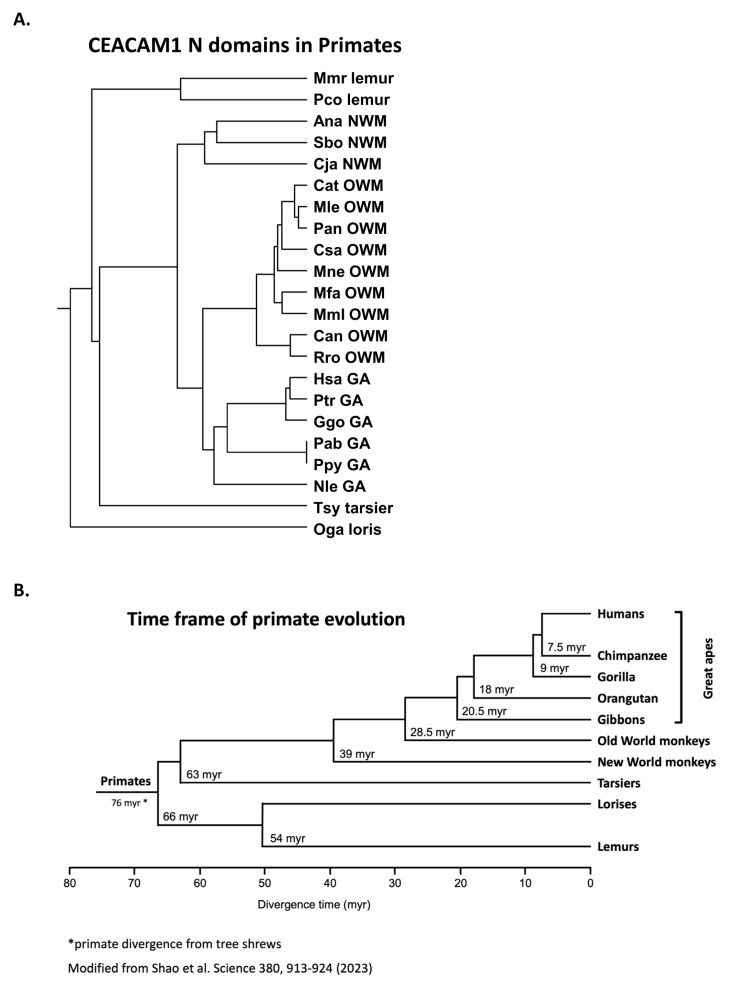
(**A**) Phylogenetic tree of amino acid sequences of mature CEACAM1 N domain of selected primates. The phylogenetic relationship of the amino acid sequences of the N domains of CEACAM1 in various primate species is shown, where the length of the line reflects the similarity of sequences using the ClustalW technique, with shorter length representing higher similarity. Species abbreviations shown in Table 1. NWM; New World monkey, OWM; Old World monkey, GA; great ape. (**B**) Time frame of primate evolution. Adapted from ref [26].

We next examined the similarity of CEACAM1 among selected primates (Table 1); close similarity is evident in the N domain amino acid sequences (Figure 2A). Among primates, the human (Hsa) CEACAM1 N domain is most similar to that of the chimpanzee (Ptr) and gorilla (Ggo) (Figure 2A). The time frame of primate evolution is shown in Figure 2B. The phylogenetic clustering of the CEACAM1 N domain does, in general, parallel that of the evolution of the primates in that they are more similar within the categories of great apes, Old World monkeys, New World monkeys, and lemurs/tarsiers [25].

### 3.3. Activating Peptides and Functionally Critical Subregions from the Human CEACAM1 N Domain Are Generally Better Conserved than the Whole Domain

**Figure 3:**
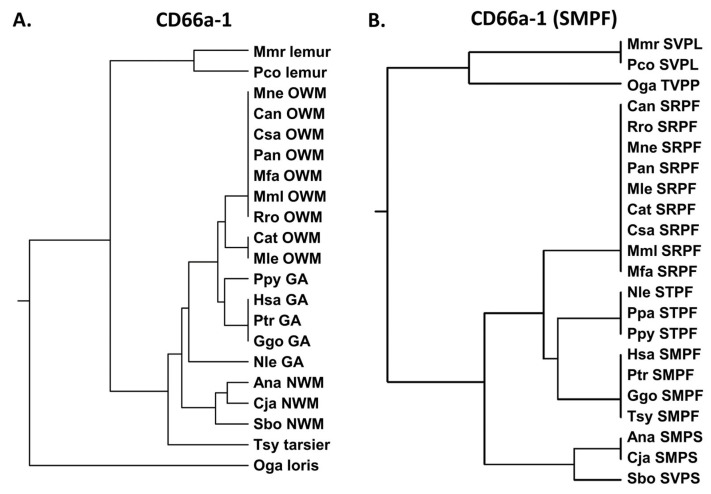
(**A**) Phylogenetic tree of amino acid sequence peptide CD66a-1 and homologs of selected primates as in Figure 2A (except for Pab). (**B**) Phylogenetic tree of amino acid sequence SMPF of CD66a-1 and homologs of selected primates as in Figure 2A (except for Pab). Corresponding amino acid sequence of each peptide is shown. Species abbreviations shown in Table 1. NWM; New World monkey, OWM; Old World monkey, GA; great ape.

Five 13- or 14-amino acid peptide sequences from the N domain of human CEACAM1 have been reported to activate human neutrophils [22,23,24] (Supplementary Tables S1–S6). These peptides (peptides CD66a-1, 2, 3, 6L, and 7) were derived from the N domain of CEACAM1 as previously described [22,24]. We examined the similarity of these five sequences in humans and the homologous peptides of other primates. In general, the active peptide CD66a-1 was more conserved among primates than the whole N domain amino acid sequences (Figure 3) in that there was more similarity of the CD66a-1 peptide in CEACAM1 among groups of primates than that of the respective CEACAM1 N domain sequences (Figure 2A). Peptide CD66a-1 in humans was identical to that in chimpanzees and gorillas, and the sequences in most of the great apes and Old World monkeys showed strong similarity (Figure 3A). The sequence of peptide CD66a-1 was more different in the New World monkeys examined (Figure 3A).

As the CEACAM family evolved, sequence changes presumably evolved that are critical to the functions the new family members developed in different species. CEACAM1, 3, 6, and 8, which are expressed on neutrophils, and CEACAM5, which is not, have different amino acid sequences. Some of the peptides homologous to the human CD66a-1 peptide in these different CEACAMs have similar activity to the peptides in CEACAM1 in that they activate human neutrophils, while some do not, and these amino acid differences presumably contribute to the different functions of the molecules evolved (Supplementary Table S2) [23,24]. We therefore examined the similarities of smaller peptide sequences that differ among these five CEACAMs and are likely critical for the functions of that active site among other species.

Previous studies suggested that the SMPF region of CD66a-1 was critical for activity based on differences in homologous peptides from different CEACAMs that were inactive (Supplementary Table S2) [23]. We therefore examined the similarity of the amino acid sequence SMPF in selected primates. Comparison of the shorter SMPF region showed identity between some great apes (human, chimpanzee, gorilla) and also the more distantly related tarsier (Figure 3B). However, the homologous peptide in other great apes (Nle, Ppa, and Ppy) all showed the substitution of T for M. While the exact contribution to activity of different parts of the 14-amino acid peptide is unclear, for peptide CD66a-1, the substitution of T for M, present in human CEACAM3, 6, 5, and 8, results in loss of activity in the human neutrophil system (Supplementary Table S2) [23]. The nonconservative substitution of M to R yielding SRPF found in all Old World monkeys analyzed suggests that these also lack activity, at least in the human system. Thus, the critical M is preserved in six of the primates examined and is replaced by T in the northern white-cheeked gibbon (Nle), as well as in ten additional gibbon species and orangutans. This suggests that an important change in this domain occurred in humans, chimpanzees, and gorillas leading to a functional change in this region of the N domain of CEACAM1. Interestingly, this sequence was conserved in the tarsier despite its early evolutionary divergence from the other primates. Among the 26 New World monkeys examined, M was present in 17 and V in 9 New World monkeys (Supplementary Figure S1). The F to S change in Ana and Cja represents a hydrophobic to polar substitution. Of the 26 New World monkeys examined, F was replaced by S in 23 and by P in 3 New World monkeys. Since an M to V substitution would be conservative, these data suggest that the original primordial sequence of CEACAM1 in this region of the New World monkey ancestor was likely SMPS.

CEACAMs 5 and 6 both have STPF sequences in the homologous region of peptide CD66a-1, whereas CEACAM8, which has not been reported to bind pathogens, has a conservative M to V substitution but also nonconservative S to A and S to F substitutions. Study of the SMPF motif has been complicated by the fact that the original CEACAM3 sequence (at the time the biologically active CEACAM1 peptides described here were identified) was reported to be STPF [2], which lacked activity in the neutrophil activation system. At present, however, the most prevalent CEACAM3 sequence in this region is known to be SMPL, suggesting the presence of alleles in the human population with alternate sequences in this region of CEACAM3. The F to L substitution is conservative and likely does not alter function in CEACAM1 or 3. Indeed, SNPs were observed with very low frequency which led to changes in the SMPL sequence in CEACAM3 (Supplementary Figure S2).

**Figure 4:**
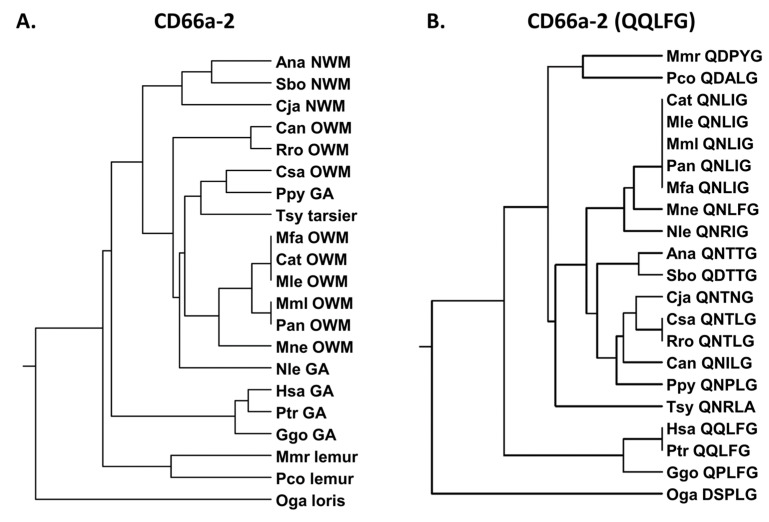
(**A**) Phylogenetic tree of amino acid sequences of CD66a-2 and homologs of selected primates as in Figure 2A (except for Pab). (**B**) Phylogenetic tree of amino acid sequence QQLFG of CD66a-2 and homologs of selected primates as in Figure 2A (except for Pab). Corresponding amino acid sequence of each peptide is shown. Species abbreviations shown in Table 1. NWM, New World monkey; OWM, Old World monkey; GA, great ape.

We next examined the human CEACAM1 peptide, CD66a-2 (Supplementary Table S3). The peptide CD66a-2 sequence showed strong similarity to chimpanzees and gorillas and less similarity to the other primates tested (Figure 4A). Earlier studies suggested that the QQLFG sequence is critical to function and the single Q to H change at position 2 results in a loss of peptide activity in the human system (Supplementary Table S3) [23]. The QQLFG sequence is identical in humans and chimpanzees (Ptr) and similar in gorillas (Ggo), but it is quite different from all of the other studied primates (Figure 4B); the Q to P substitution in the gorilla is a polar to a hydrophobic change and it may also alter the conformational rigidity of the protein. The Q to N and Q to D substitutions seen in many of the primates studied represent polar to polar and polar to negative charge substitutions, respectively. The F to I change represents a conservative hydrophobic to hydrophobic change.

**Figure 5:**
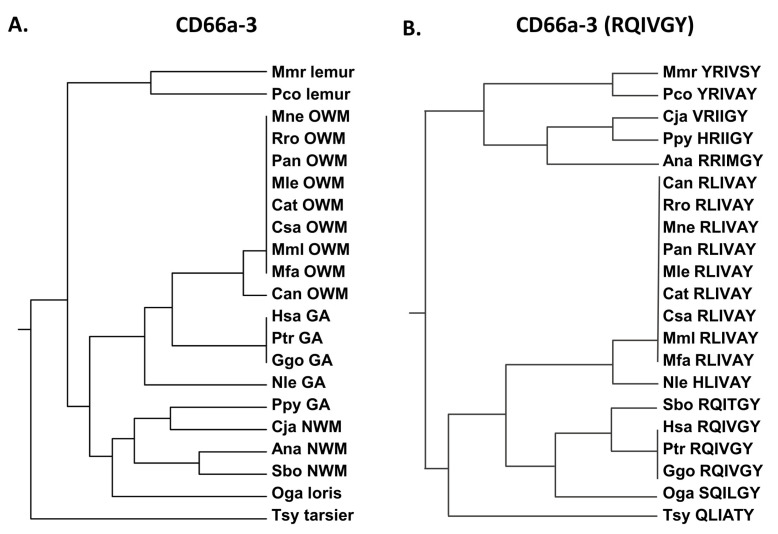
(**A**) Phylogenetic tree of amino acid sequences of CD66a-3 and homologs of selected primates as in Figure 1. (**B**) Phylogenetic tree of amino acid sequence RQIVGY of CD66a-3 and homologs of selected primates as in Figure 2A (except for Pab). Corresponding amino acid sequence of each peptide is shown. Species abbreviations shown in Table 1. NWM, New World monkey; OWM, Old World monkey; GA, great ape.

Peptide CD66a-3 in the human (Supplementary Table S4) is identical to the homologous peptide in chimpanzees and gorillas (Figure 5) and is more closely related to Old World monkeys than New World monkeys. Earlier studies of the homologous human sequences in the other CEACAMs revealed that the RQIVGY sequence, and, in particular, the RQ region, plays a critical role in peptide function (Supplementary Table S4) [23]. The NRQII and QNDTG have been reported to be involved in intercellular adhesion of CEACAM5 [27]. Sequence comparisons showed that the shorter RQIVGY sequence is again identical in humans, chimpanzees, and gorillas, but in contrast to the entire peptide sequence, it is more similar to the New World monkey (Sbo) and the more distantly related loris (Oga) than the other primates examined, including the other great apes (Nle and Ppy) (Figure 5). The Q to L and G to A substitutions present in many of the Old World monkeys examined represent a polar uncharged to hydrophobic and uncharged to hydrophobic change, respectively.

**Figure 6:**
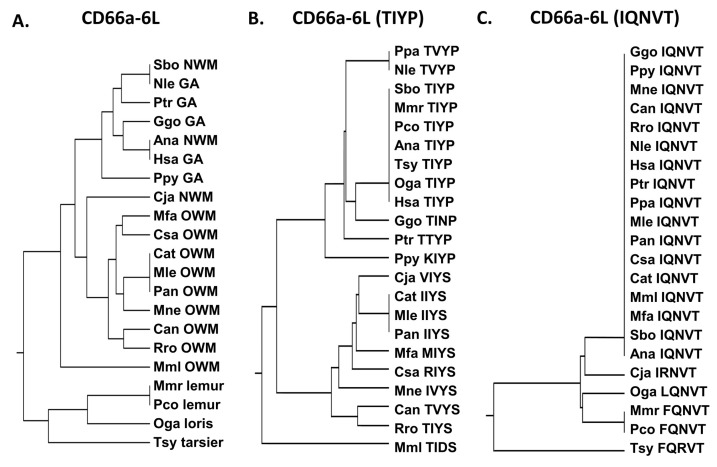
(**A**) Phylogenetic tree of amino acid sequences of CD66a-6L and homologs of selected primates as in Figure 2A (except for Pab). (**B**) Phylogenetic tree of amino acid sequence TIYP of CD66a-6L and homologs of selected primates as in Figure 2A (except for Pab). Corresponding amino acid sequence of each peptide is shown. (**C**) Phylogenetic tree of amino acid sequence IQNVT of CD66a-6L and homologs of selected primates as in Figure 2A (except for Pab). Corresponding amino acid sequence of each peptide is shown. Species abbreviations shown in Table 1. NWM, New World monkey; OWM, Old World monkey; GA, great ape.

The CD66a-6L peptide sequence is identical in humans and the New World monkey Ma’s night monkey (Ana) and most similar among the other great apes examined and the New World monkey Bolivian squirrel monkey (Sbo) (Figure 6). Several substitutions are present in the homologous peptides in CEACAM3, 5, and 8 (Supplementary Table S5). Peptide CD66a-6L is identical in CEACAM1 and 6 and there is a QN to MR change in CEACAM8; however, we do not have data on the biological activity of the corresponding CEACAM8 peptide. While the potential neutrophil activation activity of the CEACAM8, CEACAM3, and CEACAM5 CD66e-6L homologous peptides is unknown, the change in IQ to MR in CD66b-6L—representing a hydrophobic to hydrophobic change with an -SH group and a polar to charged amino acid substitution—suggests that it may be different (Supplementary Table S5).

Since the differences between CD66a-6L and the homologous peptides from CEACAMs 1, 3, 6, and 8 are only found in the TIYP and IQNVT sequences, we also examined the distribution of these sequences. The TIYP sequence was shared by humans, lemurs, lorises, and two New World monkeys examined, whereas the Old World monkeys generally formed a distinct group (Figure 6). In contrast, the IQNVT sequence was identical for all of the primates examined except one New World monkey (Cja), lemurs, lorises, and tarsiers (Figure 6), suggesting an important role of this domain in primates.

**Figure 7:**
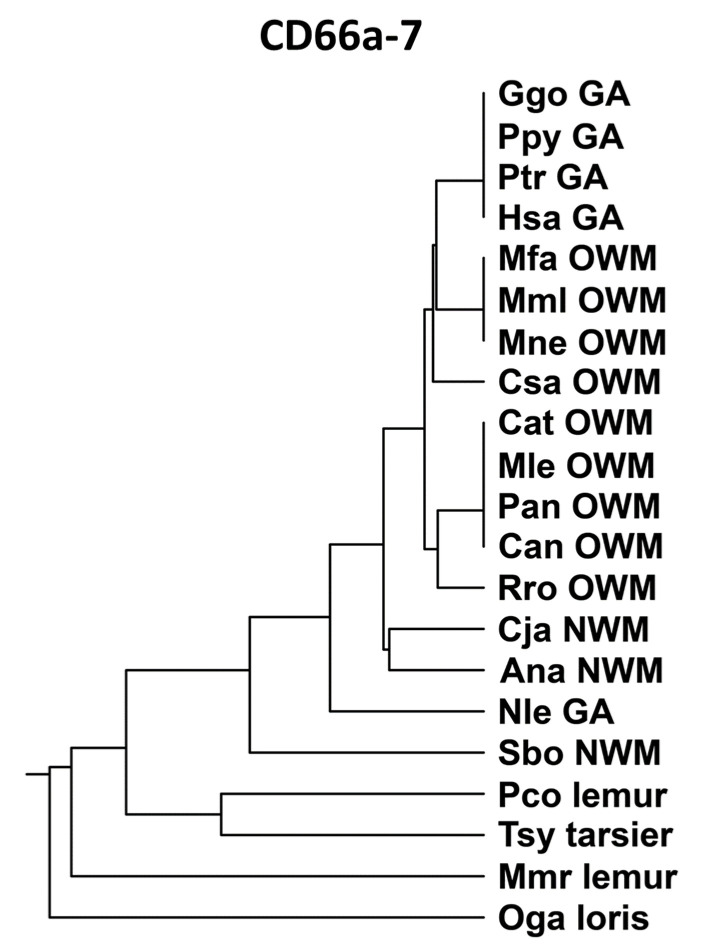
Phylogenetic tree of amino acid sequences of CD66a-7 and homologs of selected primates as in Figure 2A (except for Pab). Species abbreviations shown in Table 1. NWM, New World monkey; OWM, Old World monkey; GA, great ape.

Peptide CD66a-7 (Supplementary Table S6) was identical in humans, gorillas, and chimpanzees, and, in general, was more closely related to the homologous peptides of Old World monkeys than New World monkeys (Figure 7). The CD66a-7 sequences were identical in human CEACAMs 1, 3, 5, and 6. The homologous region of CEACAM8 had several amino acid substitutions as shown in Supplementary Table S6 and lacked biological activity in the neutrophil adhesion assay; the multiple amino acid changes precluded assessment of a specific active subdomain.

**Figure 8:**
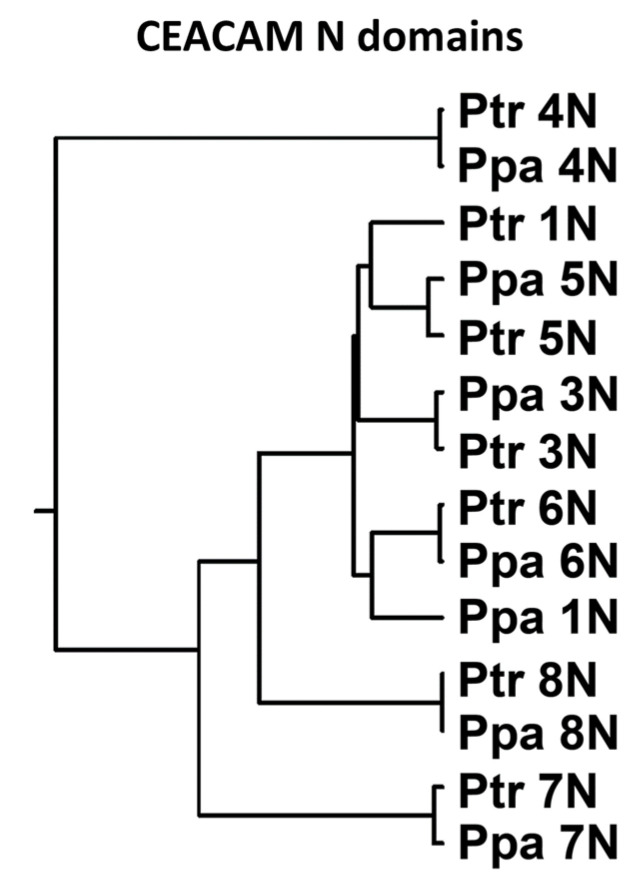
Phylogenetic tree of N exon nucleotide sequences of *CEACAM1*, *3*, *5*, *6*, *7*, and *8* of chimpanzee (Ptr) and bonobo (Ppa). The phylogenetic relationship of the nucleotide sequences of the N domain exons is shown, where the length of the line reflects the similarity of sequences using the ClustalW technique, with shorter length representing higher similarity.

Notably, an M to T amino acid substitution in the CD66a-1 peptide that leads to loss of neutrophil activating activity in humans was found in the bonobo but not the chimpanzee. This was surprising since the chimpanzee and bonobo are very closely related, being the only two species of the genus Pan. When the orthologous amino acid sequences of the CEACAM1, 3, 4, 5, 6, 7, and 8 N domains in the chimpanzee and bonobo were compared, all paired N domains were very similar or identical except for CEACAM1 (Figure 8). The N domain of CEACAM1 in chimpanzees was more similar to that in humans than to that in bonobos (Supplementary Figure S3).

### 3.4. Non-Synonymous Single Nucleotide Polymorphisms in Activating Peptides Differ Between Chimpanzees, Bonobos, and Various African Populations

**Figure 9:**
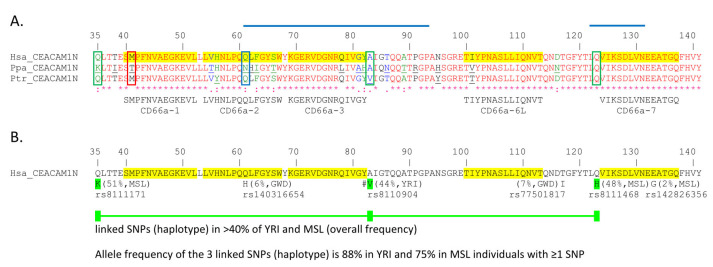
High and low frequency non-synonymous single nucleotide polymorphisms (SNPs) in human CEACAM1 N domain. (**A**) The amino acid sequences of the mature CEACAM1 N domain (leader sequences removed) from human (Hsa), bonobo (Ppa), and chimpanzee (Ptr) were aligned using the KALIGN multiple protein sequence alignment program. Amino acids deviating from the human sequence are underlined. The position of common SNPs found in certain African tribes are indicated by green boxes. A less common SNP at a position with variation in the other apes is marked in blue. A low frequency, functionally relevant SNP is boxed with red lines. The degree of conservation of the sequences is shown at the bottom of the graph [* = identical (red), : = conservative changes (green), . = less stringent conservative changes (blue)]. The location, sequence, and name of active human CEACAM1 peptides functionally tested in a granulocyte activation assay [22,23,24] are highlighted and shown below the CEACAM1 N exon amino acid sequence alignment. (**B**) High and selected low frequency non-synonymous SNPs were derived from the 1000 Genomes Project Phase 3 and Gambian Genome Variation Project; their highest population frequency in the indicated population and corresponding identifiers are indicated below the human CEACAM1N domain sequence. Amino acid numbering (1) starts at the first amino acid of the leader sequence. SNPs commonly co-inherited (haplotype) are depicted as colored boxes connected by lines. #: A to V in human CEACAM1 increases the affinity to *Helicobacter pylori* HopQ^AD-I^ adhesin 1.7-fold [3]. The regions which contain critical amino acids important for pathogen adhesin binding are highlighted by blue lines [28]. GWD, Gambian in Western Division—The Gambia, Mandinka; MSL, Mende, in Sierra Leone; YRI, Yoruba in Ibadan, Nigeria.

We also looked for differences in the other active CD66 peptides between humans, chimpanzees, and bonobos (Figure 9). The presumed active sites of peptides CD66a-2 and CD66a-3 also differed between the bonobo and chimpanzee. In bonobos, but not in chimpanzees, there is a QLF to NHI substitution in the QQLFG motif in CD66a-2 (Figure 9); this represents a polar uncharged/hydrophobic/hydrophobic to polar uncharged/positive/hydrophobic amino acid change; while the Q to N and F to I are conservative changes, the L to H represents a hydrophobic to a positively charged residue, possibly altering its activity. There is also an RQIVGY to RLIVAH change in CD66a-3 in the bonobo but not the chimpanzee (Figure 9A); the Q to L and GY to AH represent a polar to hydrophobic and an uncharged/hydrophobic to hydrophobic/positive charge change, also possibly altering its activity. A nonconservative I to T substitution was present in the TIYP region of peptide CD66a-6L of the chimpanzee and a conservative I to V substitution was present in the bonobo (Figure 9). Peptide CD66a-7 was identical in humans, chimpanzees, and bonobos.

A search for similar changes in human populations by examining the Ensembl and GnomAD databases found that an SNP representing an A > G or T > C in the opposite strand (ATG = M/ACG = T) single nucleotide variation (SNV) (rs764975765), resulting in a change from M to T in peptide CD66a-1, has been reported with a variant allele frequency (VAF) of <1%. Peptide CD66a-2 was identical in humans and chimpanzees, but a nonconservative QLF to NHI substitution was found in bonobos, who reside south of the Congo river. A mutation of A > T resulting in a Q to H substitution in QLF in peptide CD66a-2 was present with a total VAF of ~0.03% of the GnomAD database but with ~1% in humans of African descent. The Ensembl database showed a VAF for this SNP (rs140316654) with the Q to H substitution to be tribe dependent, with a VAF of ~1.1% in Africans but ~6% among the Mandinka, ~3% in the Jola, and ~0.5% among the Fula and Wolof tribes. A 133E to G change was found in CD66a-7 in 2% of the Mende (Figure 9B). In addition, a 117T to I substitution four amino acids from CD66a-6L was present in 7% of the Mandinka, and a 123Q to H change adjacent to CD66a-7 was present in 48% of the Yoruba in Ibadan and the Mende in Sierra Leone (Figure 9B).

A haplotype involving three linked, co-inherited SNPs in people of African descent, resulting in 35Q to K, 83A to V, and 123Q to H substitutions, has also been found with a frequency of ~75% in the Mende of Sierra Leone and ~88% in Yoruba tribes containing one or more of these SNPs (Figure 9). This is noteworthy in that the Q to K amino acid substitution is five amino acids from the SMPF sequence of peptide CD66a-1, the A to V substitution is adjacent to the RQIVGY sequence of peptide CD66a-3, and the Q to H substitution is adjacent to the start of the CD66a-7d (Figure 9B).

## 4. Discussion

CEACAMs are involved in multiple physiological processes and the evolution of functionally active domains should reflect this. The N domains of human CEACAMs 1, 3, 5, and 6 were found to be more closely related to each other in humans than to homologous domains in other primates. A similar finding was observed with the baboon (Pan), an Old World monkey, in that the N domains of Pan CEACAMs 1, 3, 5, and 6 were more closely related to the homologous CEACAMs in Pan than to the New World monkey, marmoset (Cja). CEACAMs 1, 3, 6, and 8 are known to functionally interact on human neutrophils, although the mechanism of this interaction is not well defined [29,30]. The high similarity of the N domains of CEACAMs 1, 3, 5, and 6 within each primate species likely reflects selective pressure of the relevant pathogens, whereby pathogen binding to CEACAM1, 5, and 6 would reflect binding to epithelial cells beneficial to the pathogen while binding to CEACAM3 could reflect its decoy function on neutrophils [31,32,33]. However, due to their anchorage via a labile glycosyl phosphatidyl inositol anchor, CEACAM5 and CEACAM6 could have a dual function as pathogen receptors and as decoy receptors (when easily shed by phospholipases) [16]. In this context, the functional relevance of the presence of CEACAM1 and CEACAM6 on granulocytes for bacterial pathogens still has to be resolved. In contrast, human CEACAM8, which does not bind known pathogens, showed higher similarity with CEACAM8 in the other primates examined; CEACAM19 exhibited a similar pattern. This finding is compatible with a lack of selective pressure from infectious agents on CEACAM8 and CEACAM19 [16]

**Figure 10:**
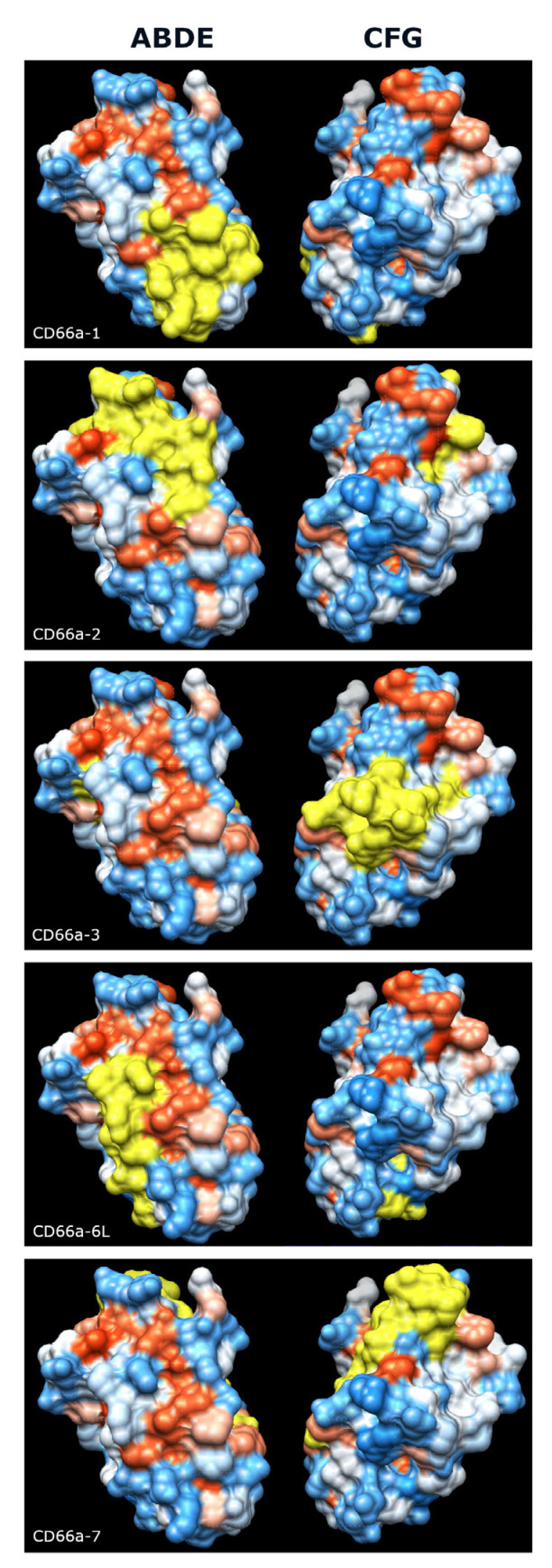
Predicted surface expression of the 5 active peptide motifs (shown in yellow) on the surface of CEACAM1. (**Left**) ABDE face, (**right**) CFG face. Electrostatic surface charge, blue indicates positive charge and red indicates negative charge. Graphics and analyses were performed with UCSF Chimera, developed by the Resource for Biocomputing, Visualization, and Informatics at the University of California, San Francisco, with support from NIH P41-GM103311 [37].

We next examined the five peptide sequences from the N domain of human CEACAM1 that have been reported to activate neutrophils [22,23,24] and found that they were more conserved among the primates examined than the respective full-length, mature N domains. As the CEACAM family evolved, sequence changes presumably occurred that are critical to the functions that new family members developed in different species. CEACAM1, 3, 6, and 8, which are expressed on neutrophils, have different amino acid sequences in these peptide regions. Some of the homologous peptides in these different CEACAMs in humans have similar activity to the peptides in CEACAM1 in that they activate neutrophils, while some do not, and these amino acid differences presumably contribute to the different functions that evolved [23,24]. Therefore, the smaller peptide sequences that differ among these five CEACAMs are likely critical for the functions of that active site among other species. Structural studies have suggested that the CFG face is the dominant site of the CEACAM1–CEACAM1 interaction [34], but three of the active peptide motifs, CD66a-1, CD66a-2, and CD66a-6L, are expressed on the ABDE face (Figure 10). These regions could be involved in the complex formation of CEACAM1 with other CEACAMs present on granulocytes. Disruption of these interactions by activating CEACAM1 peptides (as observed for certain CD66 antibodies [30]) could lead to the structural reorganization of membrane domains followed by neutrophil activation.

Previous studies suggested that the SMPF region of CD66a-1 was critical for activity (Supplementary Table S2) [23]. Comparison of the SMPF region showed identity between some great apes (human, chimpanzee, gorilla) and also the more distantly related tarsier. However, the corresponding peptide in the other great apes (Nle, Ppa, and Ppy) all showed the substitution of T for M. For peptide CD66a-1, the substitution of T for M, present in CEACAM3, 6, 5, and 8, results in a loss of activity in the human neutrophil system. The nonconservative substitution of M to R yielding SRPF in the Old World monkeys suggests that these also lack activity, at least in the human system. Thus, the critical M is preserved in six of the primates examined and is replaced by T in gibbons and orangutans, while the similarity of the full-length CD66a-1 peptide in this group appears to be more similar to the human sequence than that in most of the other primates.

A comparison of the CD66a-1 peptide between the bonobo (Ppa) and the common chimpanzee (Ptr) is particularly interesting since the M to T substitution occurs in the bonobo but not the chimpanzee. The bonobo (*Pan paniscus*) and the common chimpanzee (*Pan troglodytes*) are the two species making up the genus Pan and are the closest extant human relatives. During evolution, their ranges became separated with chimpanzees living to the north of the Congo river and bonobos living on the south of the Congo river and north of the Kasai river [18,35,36]. Since the bonobos and chimpanzees are among the most closely related primates, but evolved on different sides of the Congo river where they are exposed to a very similar climate and general environment, the M to T substitution suggests an evolutionary response to an environmental factor, most likely selective pressure from a geographically restricted pathogen, which we hypothesize to exist. CEACAM1 in chimpanzees is more similar to that in humans than bonobos. A search for a similar change in peptide CD66a-1 in human populations found that an SNV (rs764975765) resulting in the change from M to T has been reported with a low variant allele frequency (VAF) of <1%. At the time the CD66a-1 peptide described here was identified, the human sequence of CEACAM3 was found to be SMPF in this region (M to T, rs781906092, VAF < 1%); an M to I substitution has also been reported (rs1555825379, VAF < 1%). While the bonobo does not appear to have migrated far from its current range since speciation, human tribes have been much more mobile with extensive migrations within the African continent over the last 400 years, likely diluting a possible potential earlier geographic selection for various CEACAM alleles. Possible inactivating substitutions in peptides CD66a-2 and CD66a-3 are also present in bonobos but not in chimpanzees. The orthologous amino acid sequences of the CEACAM1, 3, 4, 5, 6, 7, and 8 N domains were nearly identical in chimpanzees and bonobos except for CEACAM1.

Notably, peptide CD66a-1 contains M in 17/26 and V in 9/26 New World monkeys; F was replaced by S in 23/26 and by P in 3/26 New World monkeys, suggesting that the original primordial sequence of CEACAM1 in this region in the common ancestor of haplorhini (great apes, Old World monkeys, New World monkeys, and tarsiers) was likely SMPS and was modified in Old World monkeys and New World monkeys during evolution. This suggests that an important change in this region occurred as humans, chimpanzees, and gorillas diverged from orangutans and gibbons, leading to a function change in this region of the N domain of CEACAM1. Interestingly, this sequence was conserved in tarsiers despite their early evolutionary divergence from the other primates, which suggests a conserved function in these distantly related primates.

Selective pressure from an infectious agent could also be responsible for the amino acid differences in peptides CD66a-2, CD66a-3, and CD66a-6L. Earlier studies using the neutrophil adhesion assay suggested that the QQLFG sequence in peptide CD66a-2 is critical to function and the single Q to H change results in a loss of peptide activity in the human system [23]. The QQLFG sequence is identical in humans and chimpanzees and similar in gorillas but quite different from all of the other studied primates. A nonconservative QLF to NHI substitution was found in peptide CD66a-2 in bonobos, which reside south of the Congo river. A mutation of A > T, resulting in a Q to H substitution in QLF in peptide CD66a-2, was also present in humans of African descent with a VAF of ~1% in all Africans but up to ~6% in some African tribes. The maintenance of a relatively high frequency of this allele in some African tribes despite the potential loss of some functional aspects of CEACAM1 may be due to another compensating gain such as some protection from pathogen binding to this CEACAM1 region, which is known to interact with pathogen adhesins (Figure 9) [28].

Peptide CD66a-3 in humans was more closely related to Old World monkeys than New World monkeys. Earlier studies of the human sequences in the other CEACAMs revealed that the RQIVGY sequence, and, in particular, the RQ region, plays a critical role in peptide function [23,27]. The RQIVGY sequence was identical in the human, chimpanzee, and gorilla, but in the bonobo, RLIVAH was substituted for RQIVGY. Another human haplotype of interest noted involves the coinheritance of a 77SL to RQ, 83V to A, and 103T to P in the CEACAM3 homolog of CD66a-3; this transforms the homologous region of CEACAM3 (CD66c-3) to the CEACAM1 sequence CD66a-3 [16], possibly directing CEACAM1-targeting pathogens more efficiently to the neutrophil destruction machinery via the CEACAM3 endocytic receptor.

The CD66a-6L peptide was identical in humans and the New World monkey, Ana, and most similar among the other great apes. Several substitutions are present in the homologous peptides in CEACAM3, 5, and 8; however, there are no data on the biological activity of this CEACAM8 peptide. The shorter IQVNT sequence in CD66a-6L is identical in most of the primates examined, suggesting an important role of this domain in primates, while the TIYP sequence of the peptide is much less conserved. This IQNVT sequence is identical in human CEACAMs 1, 3, and 6 but has nonconservative amino acid changes in CEACAM8, and a conservative amino acid change in CEACAM5. In addition, a 117T to I substitution four amino acids from CD66a-6L was present in the CEACAM1 N domain of ~7% of the Mandinka.

Peptide CD66a-7 was identical in humans, chimpanzees, and bonobos, and, in general, was more closely related to the homologous peptides of Old World monkeys than New World monkeys. The homologous sequences of CD66a-7 were identical in human CEACAMs 1, 3, 5, and 6. An 133E to G change was found in CD66a-7 in 2% of the Mende CEACAM1 and a 123Q to H change adjacent to CD66a-7 in 48% of the Yoruba in Ibadan and the Mende in Sierra Leone.

In addition to individual variant alleles, a haplotype involving a high frequency of coinheritance of three SNPs in people of African descent resulting in a 35Q to K, 83A to V, and 123Q to H substitution has been found with the highest VAFs between 44 and 51% in some African tribes. This is noteworthy in that the Q to K amino acid substitution is five amino acids from the SMPF sequence of peptide CD66a-1, the A to V substitution is adjacent to the RQIVGY sequence of peptide CD66a-3, and the Q to H substitution is adjacent to the start of the CD66a-7 peptide, possibly altering the tertiary structure and activity of the adjacent peptides in exchange for a potentially reduced pathogen adhesion. On the other hand, these highly selected SNPs, which probably confer an evolutionary advantage for pathogen defense, may have also been selected to not interfere with the intrinsic function of the receptor.

The comparison of bonobos to chimpanzees in the different peptide domains suggested what, in human neutrophils, would be an inactivating mutation in bonobos in peptide CD66a-1, and potentially inactivating mutations in peptide CD66a-2 (QQLFG to QNHIG), and peptide CD66a-3 (RQIVGY to RLIVAH). Peptides CD66a-1 and CD66a-2 are displayed on the ABDE face, while CD66a-3 is displayed on the CFG face. All known CEACAM1-binding pathogens have been found to bind the CFG face [28]. However, homotypic interactions between both the CFG and ABDE faces have been reported [29,34,38,39,40]. The data suggest that both the ABDE and CFG faces have physiologically important functions. No changes were noted in the putative active sites of peptide CD66a-6L (IQNVT), which is primarily on the ABDE face, or in the whole CD66a-7 peptide, which is primarily on the CFG face.

Future work could involve functional assays with neutrophils from other primates. However, the lack of such established assays and ethical constraints make such experiments difficult to perform. In addition, analysis of genome-wide association studies (GWAS) of the CEACAM1 SNPs found in humans could reveal a putative phenotypic relevance.

## 5. Conclusions

In general, the five peptide sequences from the N domain of human CEACAM1 that have been reported to activate neutrophils were more conserved among the CEACAM1 of the examined primates than the respective full-length, mature N domains. Some of the homologous peptides in different CEACAMs in humans have similar activity to the peptides in CEACAM1 in that they activate neutrophils, while some do not. Therefore, the smaller peptide sequences that differ among these five CEACAMs examined are likely critical for the functions of that active site among other species. SNPs in these peptide domains were also observed in selected human subpopulations. Taken together, the results demonstrate evolutionary differences in the CEACAM1 domain active peptides, likely reflecting the important functional roles of these regions. Given the diverse activities of CEACAM1 in humans, variant alleles in these domains might also have diverse effects in different populations.

## Figures and Tables

**Table 1:** Primate classification.

Abbreviation	Latin Name	Common Name	Classification
Ana	*Aotus nancymaae*	Ma’s night monkey	NWM
Can	*Colobus angolensis palliatus*	Black and white colobus monkey	OWM
Cat	*Cerocebus atys*	Sooty mangabey	OWM
Cja	*Callithrix jacchus*	Marmoset	NWM
Csa	*Chlorocebus sabaeus*	Green monkey	OWM
Ggo	*Gorilla gorilla*	Gorilla	GA
Hsa	*Homo sapiens*	Man	GA
Mfa	*Macaca fascicularis*	Crab-eating macaque	OWM
Mle	*Madrillus leucophaeus*	Drill	OWM
Mml	*Macaca mulatta*	Rhesus macaque	OWM
Mmr	*Microcebus murinus*	Gray mouse lemur	lemur
Mne	*Macaca nemestrina*	Pig-tailed macaque	OWM
Nle	*Nomascus leucogenys*	Northern white-cheeked gibbon	GA
Oga	*Otolemur garnettii*	Bush baby	loris
Pab	*Pongo abelii*	Sumatran orangutan	GA
Pan	*Papio* *a* *nubis*	Olive baboon	OWM
Pco	*Propithecus coquereli*	Coquerel’s sifaka	lemur
Ppa	*Pan paniscus*	Bonobo	GA
Ppy	*Pongo pygmaeus*	Bornean orangutan	GA
Ptr	*Pan troglodytes*	Chimpanzee	GA
Rro	*Rhinopithecus roxellana*	Golden snub-nosed monkey	OWM
Sbo	*Saimiri boliviensis*	Bolivian squirrel monkey	NWM
Tsy	*Tarsius syrichta*	Tarsier	tarsier

NWM; New World monkey, OWM; Old World monkey, GA; great ape.

## Data Availability

The datasets generated and/or analyzed during the current study are available in the Ensembl database (https://www.ensembl.org/Homo_sapiens/Info/Index) and the GnomADv4.1 database (https://gnomad.broadinstitute.org/news/2024-04-gnomad-v4-1/). These datasets were accessed on 17 April 2025.

## Supplementary Materials

The following supporting information can be downloaded at https://www.mdpi.com/article/10.3390/biology14121744/s1, Table S1: CEACAM1 active peptide sequences; Table S2: Neutrophil activating activity of peptide CD66a-1 and homologs; Table S3: Neutrophil activating activity of peptide CD66a-2 and homologs; Table S4: Neutrophil activating activity of peptide CD66a-3 and homologs; Table S5: Neutrophil activating activity of peptide CD66a-6L and homologs; Table S6: Neutrophil activating activity of peptide CD66a-7 and homologs; Figure S1: New World Monkey CEACAM1 N Amino Acid Sequence Alignment; Figure S2: CEACAM3 N Amino Missense SNPs in Has; Figure S3: CEACAM1 N Amino Acid Alignment of Hsa, Ptr, and Ppa.

## Abbreviations

The following abbreviations are used in this manuscript:

CEACAM	Carcinoembryonic antigen cell adhesion molecules
PSG	Pregnancy-specific glycoprotein
SNP	Single nucleotide polymorphism
SNV	Single nucleotide variation
VAF	Variant allele frequency
WGS	Whole genome sequencing
