# Supplementary material for: Evolution of CEACAM1 N Domain Biologically Active Sites in Primates
Source: Biology (Basel). 2025 Dec 5;14(12):1744. doi: 10.3390/biology14121744 (PMC12730514; doi:10.3390/biology14121744)
Supplement: Supplementary file 1 [file biology-14-01744-s001.zip › biology-3957488-supplementary.pdf]

Supplementary Table S1. CEACAM1 active peptide sequences

CD66a-1	SMPFNVAEGKEVL
CD66a-2	LVHNLPQQLFGYSW
CD66a-3	KGERVDGNRQIVGY
CD66a-6L	TIYPNASLLIQNVT
CD66a-7	VIKSDLVNEEATGQ

N domain peptides of human CEACAM1 reported to activate neutrophils [17,19].

Supplementary Table S2. Neutrophil activating activity of peptide CD66a-1 and homologs

CEACAM	PEPTIDE	SEQUENCE	ACTIVITY
CEACAM1	CD66a-1	SMPFNVAEGKEVL	+
CEACAM8	CD66b-1	**AV**P**S**N**A**AEGKEVL	-
CEACAM6	CD66c-1	S**T**PFNVAEGKEVL	-
CEACAM3*	CD66d-1*	S**T**PFNVAEGKEVL*	-
CEACAM5	CD66e-1	S**T**PFNVAEGKEVL	-

Comparison of CD66a-1 peptide of human CEACAM1 with homologous peptides of CEACAM 8, 6, 3, and 5 as described in the text (see also [18]). Amino acids underlined and shown in bold represent amino acid residues that differ from the homologous CEACAM1 peptide. Activity in

the neutrophil stimulation assay as described in references [17,18] is shown in the right column. * At the time these peptides were described, the human sequence of CEACAM3 was felt to be as shown; however, currently the dominant CEACAM3 sequence in this region is felt to be SMPLSVAEGKEVL, possibly due to variant alleles.

Supplementary Table S3. Neutrophil activating activity of peptide CD66a-2 and homologs

CEACAM	PEPTIDE	SEQUENCE	ACTIVITY
CEACAM1	CD66a-2	LVHNLPQQLFGYSW	+
CEACAM8	CD66b-2	LVHNLPQ**DPR**GY**N**W	-
CEACAM6	CD66c-2	L**A**HNLPQ**NRI**GYSW	-
CEACAM3	CD66d-2	LVHNLPQ**H**LFGYSW	-
CEACAM5	CD66e-2	LVHNLPQ**H**LFGYSW	-
		*	

Comparison of CD66a-2 peptide of human CEACAM1 with homologous peptides of CEACAM 8, 6, 3, and 5 as described in the text (see also [18]). Amino acids underlined and shown in bold represent amino acid residues that differ from the homologous CEACAM1 peptide. Activity in the neutrophil stimulation assay as described in references [17-19] is shown in the right column. * indicates the position in CD66a-2, b-2, c-2, d-2, and e-2 of Tyr34 which has been shown to be important in binding of some Opa variants by using alanine mutagenesis studies.

Supplementary Table S4. Neutrophil activating activity of peptide CD66a-3 and homologs

CEACAM	PEPTIDE	SEQUENCE	ACTIVITY
CEACAM1	CD66a-3	KGERVDGNRQIVGY	+
CEACAM8	CD66b-3	KGE**T**VD**A**NR**R**I**I**GY	-
CEACAM6	CD66c-3	KGERVDGN**SL**IVGY	-
CEACAM3	CD66d-3	KGERVDGN**SL**IVGY	-
CEACAM5	CD66e-3	KGERVDGNRQIVGY	+
		#	

Comparison of CD66a-3 peptide of human CEACAM1 with homologous peptides of CEACAM 8, 6, 3, and 5 as described in the text (see also ref [18]). Amino acids underlined and shown in bold represent amino acid residues that differ from the homologous CEACAM1 peptide. Activity in the neutrophil stimulation assay as described [18] is shown in the right column. # indicates the position in CD66a-3, b-3, c-3, d-3, and e-3 of Val39 which has been shown to be important in binding certain Opa variants and CEACAM1 homotypic adhesion by using alanine mutagenesis studies.

Supplementary Table S5. Neutrophil activating activity of peptide CD66a-6L and homologs

CEACAM	PEPTIDE	SEQUENCE	ACTIVITY
CEACAM1	CD66a-6L	TIYPNASLLIQNVT	+
CEACAM8	CD66b-6L	TIYPNASLL**MR**NVT	?
CEACAM6	CD66c-6L	TIYPNASLLIQNVT	+
CEACAM3	CD66d-6L	TIY**T**NASLLIQNVT	?
CEACAM5	CD66e-6L	**I**IYPNASLLIQN**II**	?

Amino acids underlined and shown in bold represent amino acid residues that differ from peptide CD66a-6L [19]. Peptide CD66c-6L is identical to CD66a-6L.

Supplementary Table S6. Neutrophil activating activity of peptide CD66a-7 and homologs

Activity of homologous CEACAM N domain peptides

CEACAM	PEPTIDE	SEQUENCE	ACTIVITY
CEACAM1	CD66a-7	VIKSDLVNEEATGQ	+
CEACAM8	CD66b-7	VIK**LN**L**MS**EE**V**TGQ	?
CEACAM6	CD66c-7	VIKSDLVNEEATGQ	+
CEACAM3	CD66d-7	VIKSDLVNEEATGQ	+
CEACAM5	CD66e-7	VIKSDLVNEEATGQ	+

Comparison of CD66a-7 peptide of human CEACAM1 with homologous peptides of CEACAM 8, 6, 3, and 5 as described in the text [19]. Amino acids underlined and shown in bold represent amino acid residues that differ from the homologous CEACAM1 peptide.

Supplementary Figure S1

New World monkey CEACAM1 N amino acid sequence alignment.

```
Aca_CEACAM1N    QLTVESVPSNAAEGKEVLLLTHNLPQNTTGFNWYKGERVDSTVRIMGYVISGQQITLGPAYSGRETIYPNASLLIQNVTLNDTGFYTLQVITADLVNEEVTGQFRVY
Age_CEACAM1N    QLTIESVPSNAVEGKEVLLLTHNLPQNTTGFNWYKGDRVDSTHRIIGYVTAAQKTTPGPAYSGRETIYPNGSLLIQNVTLNDTGFYTLQVIMEDLVNEEVTVQFRVY
Ahy_CEACAM1N    QLTIESVPSNAVEGKEVLLLTHNLPQNTTGFNWYKGDRVDSTHRIIGYVTAAQKTTPGPAYSGRETIYPNGSLLIQNVTLNDTGFYTLQVIMEDLVNEEVTVQFRVY
Ana_CEACAM1N    QLTIESMPSNVAEGKEVLLLTHNLPQNNTGFNWYKGESVDSTRRIMGYVTTTQQTIPGPAHSGRETIYPNASLLIQNVTLNDTGFYRLQVIMADLVNEEATGQFRVY
Apa_CEACAM1N    HLTVESVPSNAAEGKEVLLLTHNLPQNIYGFNWYKGERVDSAVRIMGYVIAAQQIIPGPAHSGRETVYPNASLLIQNVTLNDTGFYTLQVITADLVNEEVTGQFRVY
Cal_CEACAM1N    QLTIESVPSNAAEGKEVLLLTHNLPQNINGFNWYKGQSVDGTRRIIGYVIATQLTTPGPAYSSRETIYPNASLLIQNVTLNDTGFYTLQVIKVDLVNEEATGQFRVY
Cay_CEACAM1N    QLTVESMPSNAAEGKEVLLLTHNLPENSSGFNWYKGERVDSASRIIGYVIAAQRTTLGPAYSGRETIYPNASLLIQNITLNDTGFYTLQVITADLANEEATGQFRVY
Cca_CEACAM1N    QLTIESVPSNAAEGKDVLLLTHNLPQNINGFNWYKGQSVDGTRRIIGYVIATQLTTPGPAYSSQETIYPNASLLIQNVTLNDTGFYTLQVIKVDLVNEEATGQFRVY
Ccl_CEACAM1N    QLTVESMPSNAAEGKEVLLLTHNLPENSSGFNWYKGERVDSASRIIGYVIAAQRTTLGPAYSGRETIYPNASLLIQNITLNDTGFYTLQVITADLANEEATGQFRVY
Cho_CEACAM1N    QLTVESMPSNAAEGKEVLLLTHNLPENSSGFNWYKGERVDSASRIIGYVIAAQRTTLGPAYSGRETIYPNASLLIQNITLNDTGFYTLQVITADLANEEATGQFRVY
Cja_CEACAM1N    QLTTESMPSNAAEGKEVLLLTYNVPQNTNGFNWYKGERVDSTVRIIGYVIASQLTTPGPAYSGREVIYSNASLLIRNVTLNDTGFYTLQVIKADLVNEEATVQFHVY
Cml_CEACAM1N    QLTVESMPSNAAEGKEVLLLTHNLPENSSGFNWYKGERVDSASRIIGYVIAAQRTTLGPAYSGRETIYPNASLLIQNITLNDTGFYTLQVITADLANEEATGQFRVY
Lfu_CEACAM1N    QLTIESMPSNAAEGKEVLLLTHNVSQNTAGFNWYKGERVDSTIRIIGYVIATQQTTPGPAYSGRETIYPNASLLIQNVTLNDTGFYTLQVIKEDLVNEEATVQFRVY
Lla_CEACAM1N    QLTIESVPSNAAEGKEVLLLVHNLPQNTSGFNWYKGERVDGTVRIMGYVIATPQITLGPAHSGRETIYPNASLLIQNITLNDTGFYTLQVIKADLVNEEVTGQFRVY
Lni_CEACAM1N    QLTIESMPSNAAEGKEVLLLTHNVPQKTAGFNWYKGERVDSTRRIMGYVIVTQQTTPGPAHSSRETIYPNASLLIQNVTLNDTGFYTLQVIKEDLVNEEATVQFRVY
Lro_CEACAM1N    QLTTESMTSNAAEGKDVLLFTHNLPPNTAGFNWYKGERVDSTVRIIGYVIATQQTTPGPAYSGREAIYPNASLLIQNVTLNDTGFYTLQVIKEDLVNEEATVQLRVY
Pca_CEACAM1N    QLTVESMPPNAAEGKEVLLLTHNLPQNSAGFNWYKGESVDSTRRIIGYVTATQQTNRGPAYSGRETIYPNASLLIQNVTLNDTGFYTIQVITPDLTNEEATGQFRVY
Pcu_CEACAM1N    QLTVESMPPNAAEGKEVLLLTHNLPQNSAGFNWYKGESVDSTRRIIGYVTATQQTNRGPAYSGRETTYPNASLLIQNVTLNDTGFYTLQVIMPDLTNEEATGQFRVY
Pdo_CEACAM1N    QLTVESMPPNAAEGKEVLLLTHNLPQNSTGFNWYKGESVDSTRRIIGYVTATQQTNRGPAYSGRETIYPNASLLIQNVTLNDTGFYTIQVITPDLTNEEATGQFRVY
Ppi_CEACAM1N    QLTVESMPSNAAEGKEVLLLIHNLPENSSGFNWYKGGKVDSASRIIGYVIETQRTTLGPAYSGRETIYSNASLLIQNITLNDTGFYTLQVITADLTNEEVTVQFHVY
Pva_CEACAM1N    QLTVESMPSNAAEGKEVLLLTHNLPENSSGFNWYKGGKVDSASRIIGYVIATQRTTLGPAYSGRETIYPNASLLIQNITLNDTGFYTLQVITADLTNEEVTVQFRVY
Sap_CEACAM1N    QLTIESVPSNAAEGKEVLLLTHNLPQNITGFNWYKGQSVDGNRRIIGYVIATQRTTPGPAYSGRETIYPNASLLIQNVTLNDTGFYALQVIKADLVNQEATGQFRVY
Sbo_CEACAM1N    QLTIESVPSNAAEGKEVLLLARNLPQDTTGFNWHKGGSVDSTRQITGYVIATQLTTRGPAYSGRETVYPNASLLIQNVTLNDTGFYTLQVITANLVNTEATGQFRVY
Sim_CEACAM1N    QLTIESMPSNVAKGKEVLLLTHNVPQNTDGFNWYKGERVASTIRIIGYVIATQQTTPGPAHSNRETIYPNASLLIQNVTLNDTGFYTLQVIKKDLVNEEATVQFHVY
Sod_CEACAM1N    QLTIESMPSNAAEGKEVLLLTHNVPQNTTGFNWYKGERVDGTVRIIGYVIATQQTTPGPAHSSRETIYPNASLLIQNIILNDTGFYTLQVIKGDLVNEEATVQFRVY
Soe_CEACAM1N    QLTIESMPSNAAEGKEVLLLTHNVPQNTTGFNWYKGERVDGTVRIIGYVIATQQTTPGPAHSSRETIYPNASLLIQNIILNDTGFYTLQVIKGDLVNEEATVQFRVY
                :** **:..*..:**:***:  *:.  .   ****:**  *  .   :* ***        ***:*.:*. *.*.****:*: ******* :***   :*.* *.* *::**
```

The one-letter code mature CEACAM1 N domain amino acid sequences (leader sequences were removed) from 26 New World monkey species were aligned using the KALIGN multiple protein sequence alignment program. *CEACAM1* N exon sequences were retrieved from the NCBI database using the blastn suite. The CEACAM1 N exon sequences could be confidently discriminated from closely related *CEACAM5* and *CEACAM6* N exon sequences in being located at an appropriate distance from ITIM motif-encoding exons specific for *CEACAM1* genes. The functionally important four amino acid sequence that corresponds to the region of the CD66a-1 peptide from the human CEACAM1 N domain sequence is highlighted by a red box. The most prominent sequence SMPS among the 26 species (13/26) are marked in gray. Please refer to Table 1 and Supplementary Figure 1 for the three-letter code abbreviation of the species names. The conservation of amino acids at a given position is indicated by the following color and symbol code (below): red, identity (*); green, conservative changes (:); blue, less conservative changes (.); black, no conservation ( ).

Supplementary Figure S2

CEACAM3 N amino missense SNPs in Hsa (Ensembl access 23.2.2025; GomonAD4.1)

```
                       10        20        30        40        50        60        70        80        90       100
                        |         |         |         |         |         |         |         |         |         |
Hsa_CEACAM3N   KLTIESMPLSVAEGKEVLLLVHNLPQHLFGYSWYKGERVDGNSLIVGYVIGTQQATPGAAYSGRETIYTNASLLIQNVTQNDIGFYTLQVIKSDLVNEEATGQFHVY
Ptr_CEACAM3N   QLTIESTPFNVAEGKEVLLLVHNLPQHLFGYTWYKGERVDGNSLIVAYAVGIQQATPGAAYSGRETIYPNASLLIQNVTQNDIGFYTLQVIKSDLVNEEATGQFHVY
Ppa_CEACAM3N   QLTIESTPFNVAEGKEVLLLVHNLPQHLFGYTWYKGERVDGNSLIVAYAVGIQQATPGAAYSGQETIYPNASLLIQNVTQNDIGFYTLQVIKSDLVNEEATGQFHVY
               :***** *:.*********************:**************.*.:* ***********:****.**************************************

Hsa_SNPs       QI  KFTTFNG GR K   V  SMLHYI   T HE K     TQ  E IV I    SRPTNGSQ K HPSE P FRSF HKGTV FP H K  H GK GTIERL
                     IS     E            L       R G         A AK      LE S  R            K   R     H             S
                      L                  Q                      T            C     MSL,36%    E                  GWJ,10%
                                                          MSL,36%            D
                                                               MSL,36%       V
                                                                         GWD,18%
                                                                           GWD,14%

                  M->T rs781906092 VAF in Latino (amr) 4.472e-05 (2/44720)
                  M->I rs1555825379 VAF in non-Finnish European (nfe) 3.597e-06 (4/1112006)
```

1000 Genomes Project Phase 3 allele frequencies for African tribes
Non-highlighted -> highest population minor allele frequency(MAF) <0.01

Mende in Sierra Leone (MSL)
Gambian in Western Division – Mandinka (GWD)
Gambian in Western Division – Jola (GWJ)
Yoruba in Ibadan, Nigeria (YRI)
South Asian (SAS)

All high frequency SNPs but the SNP found in GWJ adjust the CEACAM3N sequence to that of CEACAM1N

Supplementary Figure S3

CEACAM1 N Amino Acid Alignment of Hsa, Ptr, and Ppa

```
                        10        20        30        40        50        60        70        80        90        100
                         |         |         |         |         |         |         |         |         |         |
Hsa_CEACAM1N    QLTTESMPFNVAEGKEVLLLVHNLPQQLFGYSWYKGERVDGNRQIVGYAIGTQQATPGPANSGRETIYPNASLLIQNVTQNDTGFYTLQVIKSDLVNEEATGQFHVY
Ptr_CEACAM1N    QLTTESMPFNVAEGKEVLLLVYNLPQQLFGYSWYKGERVDGNRQIVGYVIGTQQATPGPAYSGRETTYPNASLLIQNVTQNDTGFYTLQVIKSDLVNEEATGQFHVY
Ppa_CEACAM1N    KLTIESTPFNVAEGKEVLLLTHNLPQNHIGYTWYKGERVDGNRLIVAHAIQNQQTTRGPAHSGRETVYPNASLLIQNVTQNNTGFYTLQVIKSDLVNEEATGQFHVY
                :** ** *************.:****: :**:*********** **.:.* .**:* *** ***** **************:*************************
```

```
                        10        20        30        40        50        60        70        80        90        100
                         |         |         |         |         |         |         |         |         |         |
Hsa_CEACAM1N    QLTTESMPFNVAEGKEVLLLVHNLPQQLFGYSWYKGERVDGNRQIVGYAIGTQQATPGPANSGRETIYPNASLLIQNVTQNDTGFYTLQVIKSDLVNEEATGQFHVY
Ptr_CEACAM1N    QLTTESMPFNVAEGKEVLLLVYNLPQQLFGYSWYKGERVDGNRQIVGYVIGTQQATPGPAYSGRETTYPNASLLIQNVTQNDTGFYTLQVIKSDLVNEEATGQFHVY
                *********************:**************************.*********** ***** ****************************************
```

Alignment data :
Alignment length : 107
Identity (*) : 103 is 96.26 %

```
                        10        20        30        40        50        60        70        80        90        100
                         |         |         |         |         |         |         |         |         |         |
Ppa_CEACAM1N    KLTIESTPFNVAEGKEVLLLTHNLPQNHIGYTWYKGERVDGNRLIVAHAIQNQQTTRGPAHSGRETVYPNASLLIQNVTQNNTGFYTLQVIKSDLVNEEATGQFHVY
Ptr_CEACAM1N    QLTTESMPFNVAEGKEVLLLVYNLPQQLFGYSWYKGERVDGNRQIVGYVIGTQQATPGPAYSGRETTYPNASLLIQNVTQNDTGFYTLQVIKSDLVNEEATGQFHVY
                :** ** *************.:****: :**:*********** **.:.* .**:* ***:*****.**************:*************************
```

Alignment data :
Alignment length : 107
Identity (*) : 87 is 81.31 %

```
                        10        20        30        40        50        60        70        80        90        100
                         |         |         |         |         |         |         |         |         |         |
Hsa_CEACAM3N    KLTIESMPLSVAEGKEVLLLVHNLPQHLFGYSWYKGERVDGNSLIVGYVIGTQQATPGAAYSGRETIYTNASLLIQNVTQNDIGFYTLQVIKSDLVNEEATGQFHVY
Ptr_CEACAM3N    QLTIESTPFNVAEGKEVLLLVHNLPQHLFGYTWYKGERVDGNSLIVAYAVGIQQATPGAAYSGRETIYPNASLLIQNVTQNDIGFYTLQVIKSDLVNEEATGQFHVY
Ppa_CEACAM3N    QLTIESTPFNVAEGKEVLLLVHNLPQHLFGYTWYKGERVDGNSLIVAYAVGIQQATPGAAYSGQETIYPNASLLIQNVTQNDIGFYTLQVIKSDLVNEEATGQFHVY
                :***** *:.*********************:**************.*.:* ***********:****.**************************************
```

```
                        10        20        30        40        50        60        70        80        90        100
                         |         |         |         |         |         |         |         |         |         |
```

```
Hsa_CEACAM4N    QFTIEALPSSAAEGKDVLLLACNISETIQAYYWHKGKTAEGSPLIAGYITDIQANIPGAAYSGRETVYPNGSLLFQNITLEDAGSYTLRTINASYDSDQATGQLHVH
Ptr_CEACAM4N    QFTIEALPSSAAEGKDVLLLACNISETIQAYYWHKGKTAEGSPLIAGYITDIQANIPGAAYSGRETVYPNGSLLFQNITLEDAGSYTLRTINASYDSDQATGQLHVH
Ppa_CEACAM4N    QFTIEALPSSAAEGKDVLLLACNISETIQAYYWHKGKTAEGSPLIAGYITDIQANIPGAAYSGRETVYPNGSLLFQNITLEDAGSYTLRTINASYDSDQATGQLHVH
                ***********************************************************************************************************

                        10        20        30        40        50        60        70        80        90       100
                         |         |         |         |         |         |         |         |         |         |
Hsa_CEACAM5N    KLTIESTPFNVAEGKEVLLLVHNLPQHLFGYSWYKGERVDGNRQIIGYVIGTQQATPGPAYSGREIIYPNASLLIQNIIQNDTGFYTLHVIKSDLVNEEATGQFRVY
Ptr_CEACAM5N    KLTIESTPFNVAEGKEVLLLVHNLPQHLFGYVWYKGERVDANHQIIGYVIGTQQATPGPAYSGRETVYPNASLLIQNIIQNDTGFYTLHIIKSDLVNEEATGQFRVY
Ppa_CEACAM5N    KLTIESTPFNVAEGKEVLLLVHNLPQHLFGYVWYKGERVDGNHQIIGYVIGTQQATPGPAYSSRETIYPNASLLIQNIIQNDTGFYTLHIIKSDLVNEEATGQFRVY
                ******************************* ********.*:*******************.** :**********************:*****************

                        10        20        30        40        50        60        70        80        90       100
                         |         |         |         |         |         |         |         |         |         |
Hsa_CEACAM6N    KLTIESTPFNVAEGKEVLLLAHNLPQNRIGYSWYKGERVDGNSLIVGYVIGTQQATPGPAYSGRETIYPNASLLIQNVTQNDTGFYTLQVIKSDLVNEEATGQFHVY
Ptr_CEACAM6N    KLTIESTPFNVAEGKEVLLLAHNLPQNRIGYSWYKGERVDGNRLIVGYVIGTQQATPGPAYSGRETIYPNASLLIQNVTQNDTGFYTLQVIKSDLVNEEATGQFHVY
Ppa_CEACAM6N    KLTIESTPFNVAEGKEVLLLAHNLPQNRIGYSWYKGERVDGNRLIVGYVIGTQQATPGPAYSGRETIYPNASLLIQNVTQNDTGFYTLQVIKSDLVNEEATGQFHVY
                ****************************************** ****************************************************************

                        10        20        30        40        50        60        70        80        90       100
                         |         |         |         |         |         |         |         |         |         |
Hsa_CEACAM7N    QTNIDVVPFNVAEGKEVLLVVHNESQNLYGYNWYKGERVHANYRIIGYVKNISQENAPGPAHNGRETIYPNGTLLIQNVTHNDAGFYTLHVIKENLVNEEVTRQFYVF
Ptr_CEACAM7N    QTNIDVVPFNVAEGKEVLLVVHNESQNLYGYNWYKGERVHANYRIIGYVKNRSQENAPGPAHNGRETIYPNGTLLIQNVTHNDAGIYTLHVIKENLVNEEVTSQFYVF
Ppa_CEACAM7N    QTNIDVVPFNVAEGKEVLLVVHNESQNLYGYNWYKGERVHANYRIIGYVKNISQENAPGPAHNGRETIYPNGTLLIQNVTHNDAGIYTLQVIKENLVNEEVTSQFYVF
                *************************************************** *********************************:***:************ *****

                        10        20        30        40        50        60        70        80        90       100
                         |         |         |         |         |         |         |         |         |         |
Hsa_CEACAM8N    QLTIEAVPSNAAEGKEVLLLVHNLPQDPRGYNWYKGETVDANRRIIGYVISNQQITPGPAYSNRETIYPNASLLMRNVTRNDTGSYTLQVIKLNLMSEEVTGQFSVH
Ptr_CEACAM8N    QLTIEAVPSNAAEGKEVLLLVHNLPQDPRGYNWYKGETVDANRRIIGYVISNQQITPGPAYSNRETIYPNASLLMRNVTRNDTGSYTLQVIKLNLMSEEVTGQFSVH
Ppa_CEACAM8N    QLTIEAVPSNAAEGKEVLLLVHNLPQDPRGYNWYKGETVDANRRIIGYVISNQQITPGPAYSNRETIYPNASLLMRNVTRNDTGSYTLQVIKLNLMSEEVTGQFSVH
                ***********************************************************************************************************

                        10        20        30        40        50        60        70        80        90       100       110
                         |         |         |         |         |         |         |         |         |         |         |
Hsa_CEACAM19N   QAALYIQKIPEQPQKNQDLLLSVQGVPDTFQDFNWYLGEETYGGTRLFTYIPGIQRPQRDGSAMGQRDIVGFPNGSMLLRRAQPTDSGTYQVAITINSEWTMKAKTEVQA
Ptr_CEACAM19N   QAALYIQKIPEQPQKNQDLLLSVQGVPDTFQDFNWYLGEETYGGTRLFTYIPGIQRPQRDGSAMGQRDIVGFPNGSMLLRRAQPTDSGTYQVAITINSEWTMKAKTEVQA
Ppa_CEACAM19N   QAALYIQKIPEQPQKNQDLLLSVQGVPDTFQDFNWYLGEETYGGTRLFTYIPGIQRPQRDGSAMGQRDIVGFPNGSMLLRRAQPTDSGTYQVAITINSEWTMKAKTEVQA
                **************************************************************************************************************
```
